# Time to virologic failure for patients taking their first antiretroviral regimen and the subsequent resistance profiles

**DOI:** 10.7448/IAS.17.4.19757

**Published:** 2014-11-02

**Authors:** Frederic Crouzat, Anita Benoit, Colin Kovacs, Graham Smith, Nathan Taback, Ina Sandler, Jason Brunetta, Benny Chang, Barry Merkley, David Tilley, David Fletcher, Dipen Kalaria, Mona Loutfy

**Affiliations:** 1 Infectious Diseases, Maple Leaf Medical Clinic, Toronto, Canada; 2 Women's College Research Institute, Women's College Hospital, Toronto, Canada; 3 Department of Statistical Sciences, University of Toronto, Toronto, Canada; 4 Data Management, Maple Leaf Medical Clinic, Toronto, Canada; 5 Virology and Vaccines, Janssen Inc., Toronto, Canada

## Abstract

**Introduction:**

The resistance profiles of first-line antiretroviral therapy (ART) regimens after virologic failure have yet to be studied in a clinic setting in the modern treatment era. Time to virologic failure among three standard first-line regimens and the resistance profiles of these failures were compared.

**Materials and Methods:**

All HIV-positive persons aged 16 and over starting a three-drug first-line ART regimen were retrospectively identified at a Toronto community clinic (1 January 2006–1 January 2013). The regimens included a backbone of two NRTIs and a third agent; a PI, an NNRTI, or an II. Patients must have been on treatment for at least 14 days and have at least one VL test within 6 months after starting treatment. The primary outcome was virologic failure defined as either: no suppression by 6 months, or after suppression, two consecutive, detectable VL200 copies/mL at least 14 days apart or one VL>200 copies/mL. Time to failure was compared using a proportional hazards model adjusting for demographic and clinical factors. Resistance profiles of NRTIs and third agents are described in patients with virologic failure who had both baseline and virologic failure genotypes.

**Results:**

Six hundred sixty patients (93% male) were included with a mean age of 38.9 and a median follow-up period of 35.3 (32.2–39.3) months. Distribution of third agent use was: PI 37.3% (n=246), NNRTI 55.9% (n=369) and II 6.8% (n=45). Virologic failures occurred in 81/246 (33%) with PI, 87/369 (24%) with NNRTI and 11/45 (24%) with II. Compare to PIs, time to failure was longer with NNRTIs (p=0.0013) and similar for IIs (p=0.1562). No evidence that failure with NNRTIs was different from IIs (p=0.9139). Of the 660 patients, 567 (86%) had a baseline genotype. Of the 567 patients, 179 had virological failure. Of the 179, 145(81%) had a baseline genotype and only 37 (21%) had both a baseline and follow-up genotype. Upon failure, emerging ART resistance was rare. No new PI or II mutations were identified and one new NNRTI (Y181C) mutation was identified. Three patients taking PI-based regimens developed NRTI mutations (M184V, M184I, T215Y).

**Conclusions:**

Time to virologic failure was significantly greater in the NNRTI group compared to the PI group. If failure did occur, ART resistance rarely developed with no PI mutations but a few NRTI mutations in those taking PI-based regimens, and NNRTI mutations in those taking NNRTI-based regimen.

